# Isolation, characterization and functional analysis of a bacteriophage targeting *Culex pipiens pallens* resistance-associated *Aeromonas hydrophila*

**DOI:** 10.1186/s13071-024-06281-x

**Published:** 2024-05-15

**Authors:** Jinze Li, Jiajia Du, Guangshuo Ding, Wenxing Zhang, Yinghui Zhou, Yidan Xu, Dan Zhou, Yan Sun, Xiaoqiu Liu, Bo Shen

**Affiliations:** 1 Department of Pathogen Biology, Nanjing Medical University, Nanjing, Jiangsu China; 2 Department of Pathogen Biology, China Medical University, Shenyang, China

**Keywords:** *Culex pipiens pallens*, Deltamethrin resistance, *Aeromonas hydrophila*, Bacteriophage, Gut microbiota

## Abstract

**Background:**

*Culex pipiens pallens* is a well-known mosquito vector for several diseases. Deltamethrin, a commonly used pyrethroid insecticide, has been frequently applied to manage adult *Cx. pipiens pallens*. However, mosquitoes can develop resistance to these insecticides as a result of insecticide misuse and, therefore, it is crucial to identify novel methods to control insecticide resistance. The relationship between commensal bacteria and vector resistance has been recently recognized. Bacteriophages (= phages) are effective tools by which to control insect commensal bacteria, but there have as yet been no studies using phages on adult mosquitoes. In this study, we isolated an *Aeromonas* phage vB AhM-LH that specifically targets resistance-associated symbiotic bacteria in mosquitoes. We investigated the impact of *Aeromonas* phage vB AhM-LH in an abundance of *Aeromonas hydrophila* in the gut of *Cx. pipiens pallens* and its effect on the status of deltamethrin resistance.

**Methods:**

Phages were isolated on double-layer agar plates and their biological properties analyzed. Phage morphology was observed by transmission electron microscopy (TEM) after negative staining. The phage was then introduced into the mosquito intestines via oral feeding. The inhibitory effect of *Aeromonas* phage vB AhM-LH on *Aeromonas hydrophila* in mosquito intestines was assessed through quantitative real-time PCR analysis. Deltamethrin resistance of mosquitoes was assessed using WHO bottle bioassays.

**Results:**

An Aeromonas phage vB AhM-LH was isolated from sewage and identified as belonging to the *Myoviridae* family in the order *Caudovirales* using TEM. Based on biological characteristics analysis and in vitro antibacterial experiments, *Aeromonas* phage vB AhM-LH was observed to exhibit excellent stability and effective bactericidal activity. Sequencing revealed that the *Aeromonas* phage vB AhM-LH genome comprises 43,663 bp (51.6% CG content) with 81 predicted open reading frames. No integrase-related gene was detected in the vB AH-LH genome, which marked it as a potential biological antibacterial. Finally, we found that *Aeromonas* phage vB AhM-LH could significantly reduce deltamethrin resistance in *Cx. pipiens pallens*, in both the laboratory and field settings, by decreasing the abundance of *Aeromonas hydrophila* in their midgut.

**Conclusions:**

Our findings demonstrate that *Aeromonas* phage vB AhM-LH could effectively modulate commensal bacteria *Aeromonas hydrophila* in adult mosquitoes, thus representing a promising strategy to mitigate mosquito vector resistance.

**Graphical Abstract:**

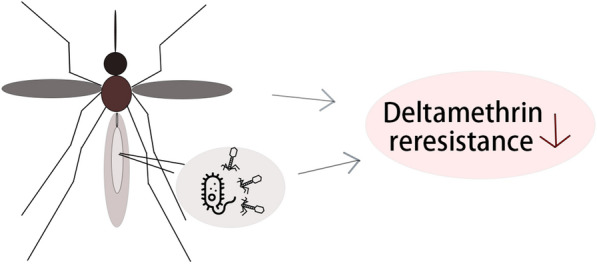

**Supplementary Information:**

The online version contains supplementary material available at 10.1186/s13071-024-06281-x.

## Background

*Culex pipiens pallens* is a significant vector for various viruses, including West Nile Virus, Usutu virus, Rift Valley fever virus, and Japanese encephalitis virus [1, 2]. One of the more useful methods of vector control during disease outbreaks is adult control, which effectively reduces disease transmission by decreasing the population of disease-carrying females as well as their overall reproductive capacity [3]. Pyrethroid insecticides are commonly used to control *Cx. pipiens pallens* adults [4], but the widespread use of these insecticides has led to the development of resistance in mosquitoes [5]. Therefore, it is necessary to develop new control strategies that target vector insecticide resistance.

In recent years, studies have indicated the involvement of intestinal commensal bacteria in regulating mosquito resistance. There are notable disparities in the composition of intestinal microbial communities between insecticide-sensitive and -resistant mosquitoes. *Asiaia* and *Serratia* bacteria have been observed to contribute to the development of mosquito sensitivity to deltamethrin; for example, *Serratia oryzae* can significantly enhance the resistance of *Aedes albopictus* to deltamethrin by upregulating the expression of metabolic detoxification genes [6, 7]. Our previous study identified *Aeromonas hydrophila* as the predominant bacterial species in the intestinal tract of a deltamethrin-resistant strain of *Cx. pipiens pallens* and noted that its abundance was significantly higher than that in the sensitive strain [8]. Further investigations demonstrated that *Aeromonas hydrophila* can directly degrade deltamethrin and increase the activity of related metabolic detoxification enzymes, thereby enhancing the resistance of *Cx. pipiens pallens* to deltamethrin [9].

Antibiotics are commonly used to control mosquito gut microbes; however, it is important to note that most of the commensal bacteria in the mosquito gut are antibiotic resistant [10] and that the use of antibiotics can disrupt the microbial balance, making it challenging to eliminate the natural microbiota [10]. Additionally, antibiotics can have various effects on the host, such as influencing host immunity and metabolism, inhibiting eukaryotic translation and altering mitochondrial function [11–13]. In the field of invertebrate research, bacteriophages (commonly known as phages) are widely utilized as alternatives to antibiotics [14]; for example, bacteriophage Y12Pw is effective in reducing the numbers of *Pseudomonas aeruginosa* in the complex intestinal environment of housefly larvae [15]. It is important to note that the proliferation of phages can also have an impact on the development of housefly larvae [16]. The authors of one study reported that the addition of phages to water resulted in a decrease in the presence of bacteria that support larval development and survival, and that this, in turn, can negatively affect the health of mosquito larvae [17]. Nevertheless, the consequences of phage-mediated changes in the intestinal microbiota of adults are incompletely understood.

In the present study, we isolated a lytic *Aeromonas* phage vB AhM-LH from a river in Nanjing, China. The host bacterium of *Aeromonas* phage vB AhM-LH is *Aeromonas hydrophila* AH3.5, which is a strain isolated from deltamethrin-resistant *Cx. pipiens pallens*. The specific morphological classification of *Aeromonas* phage vB AhM-LH was carried out using transmission electron microscopy (TEM), and its biological properties were determined.

Through whole-genome sequencing, we obtained the complete genome sequence of the phage and conducted comparative genomic analysis. Finally, we verified the bacteriostatic effect of the phage in vivo in laboratory and field mosquitoes, as well as its impact on the deltamethrin-resistant phenotype of females.

## Methods

### Mosquito species

*Culex pipiens pallens* mosquitoes were collected in Tangkou, Shandong Province, China.

The median lethal concentration (LC50) of deltamethrin to larvae was determined to be 0.04 mg/l using the WHO insecticide susceptibility test. The deltamethrin-sensitive strains (DS) had not been previously exposed to any insecticides. The DS mosquitoes were subjected to deltamethrin treatment (45423; Sigma-Aldrich, St Louis, MO, USA) using the WHO larval dipping method at the LC50 concentration to establish a deltamethrin-resistant strain (DR) over approximately 33 generations; the DR strain had an LC50 of 1.2 mg/l [18, 19].

Larvae were raised in dechlorinated water and fed daily with mouse chow powder (1010019; Xietong Pharmaceutical Bioengineering Co., Ltd., Nanjing, China). Adult mosquitoes were provided with a 5% glucose solution, which was replenished every 24 h. The rearing conditions included a room temperature of 27 °C, a light/dark cycle of 14/10 h and a relative humidity of 70%. Institute of Cancer Research (ICR) mouse blood was used to passage the mosquitoes, using mice obtained from the Animal Core Facility of Nanjing Medical University [20]. The animal experiment protocols were approved by the Ethics Review Committee of Nanjing Medical University (No. IACUC-1812047).

To further verify the potential application value of *Aeromonas* phage vB AhM-LH, field samples of *Cx. pipiens pallens* larvae were collected from ditches near a community in Nanjing, Jiangsu Province, China. Larvae in the water samples collected from the breeding site were transferred to the laboratory and retained for further experiments [21]. To identify the species of mosquitoes collected from the field, DNA was amplified from the mosquitoes after emergence using the invertebrate cytochrome* c* oxidase subunit 1 (COI) universal primers. PCR amplification verification was performed in a PCR total reaction volume of 25 μl consisting of 12.5 μl of 2× Master Mix (Vazyme Company, Nanjing, China), 9.5 μl of sterile water, 1 μl of total DNA template (approx. 400 ng) and 1 μl each of the upstream and downstream primers (10 μM). The PCR reaction conditions included an initial denaturation step at 95 °C for 3 min; followed by 30 cycles of denaturation at 95 °C for 15 s, annealing at 56 °C for 15 s and extension at 72 °C for 60 s; with a final extension step at 72 °C for 5 min. The forward primer used was LOC1490 (5ʹ-GGTCAACAAATCATAAAGATATTGG-3ʹ) and the reverse primer used was HCO2198 (5ʹ-TAAACTTCAGGGTGACCAAAAAATCA-3). The amplified product was sent to General Biotech Company (Anhui, China) for sequencing, and the resulting gene sequence was searched in the NCBI database for comparison [22].

#### Isolation of *Aeromonas hydrophila* AH3.5

*Aeromonas hydrophila* AH3.5 was isolated from the intestinal tract of DR female *Cx. pipiens pallens*. Female mosquitoes were rinsed in 75% ethanol followed by sterile phosphate-buffered saline (PBS) to remove residual ethanol on the surface of the body. The midguts were then excised, ground and inoculated onto sterile Luria–Bertani (LB) agar plates using a three-zone marking method. Colonies that formed were purified by picking a single colony for expansion in liquid LB culture medium. This process was repeated 3 times to obtain stable and pure bacterial strains. A bacterial genome extraction kit from Tiangen Biochemical Technology Co., Ltd. (Beijing, China) was utilized, along with bacterial universal primers [Eub(27)F/Eub(1492)R] for PCR amplification. The amplified products were then forwarded to General Biotech Company for strain identification.

The isolated bacteria were mixed with 40% glycerol and stored at − 80 °C in a freezer in the insect room of the Department of Pathogen Biology, Nanjing Medical University for long-term preservation [9].

### Phage isolation, purification and transmission electron microscopy

Bacteriophages were isolated from Nanjing sewage using the double-layer agar plate method [23]. Larger single plaques were selected and inoculated into medium containing *Aeromonas hydrophila* AH3.5 in the logarithmic growth phase (OD_600_ = 0.1). The mixture was placed on a shaking table and shaken at 30 °C for 2 h to amplify the phage, then the double-layer agar plate was spread again; the process was repeated another 3 times to obtain a single phage [24]. The phage lysate was then filtered through a 0.22-μm needle filter and concentrated using an ultrafiltration unit with a 100-kDa cutoff (UFC9100; Sigma-Aldrich) [25]. Finally, the purified phage was obtained through cesium chloride gradient centrifugation (V900481; Sigma-Aldrich) [26].

The negative staining technique was used to prepare samples for TEM. A total of 10 μl of the purified phage solution was dropped onto a copper mesh and allowed to stand for 10 min [27]. The excess liquid was then absorbed, and the copper mesh was placed upside down on 10 μl of 2% phosphotungstic acid-negative stain solution (G1870; Solarbio, Beijing, China). After allowing for the excess dye to be absorbed for 1 min, we left the sample to dry at room temperature. The morphology of the sample was observed in a transmission electron microscope (FEl Tecnai Spiritt) at 120 kV and 12,000× magnification [27].

### Phage biological properties

*Aeromonas hydrophila* AH3.5 in the early logarithmic stage (OD_600_ = 0.1) was mixed with phages at different multiplicities of infection (MOI; 100, 10, 1, 0.1, 0.01 and 0.001), and the mixture was shaken at 30 °C for 5 h. The phage titer was determined on agar plates, and the group with the highest titer was considered to have the optimal MOI. Each group consisted of three biological replicates and three technical replicates. *Aeromonas hydrophila* AH3.5 and *Aeromonas* phage vB AhM-LH were mixed according to the optimal MOI of 10. The mixture was incubated at room temperature for 10 min, then centrifuged at 10,000 *g* for 1 min, following which the supernatant was discarded and the mixture resuspended in sterile SM buffer (PH1845; Overview Phygene, Fuzhou, China); this process was repeated 3 times. The mixture was then inoculated into 10 ml of LB culture medium and incubated at 30 °C with shaking for 80 min. The sample titer was measured every 10 min using a double-layer agar plate, with three measurements taken at each time point. One-step growth curves were drawn based on the obtained results. The concentration of *Aeromonas* phage vB AhM-LH was adjusted to 10^8^ plaque-forming units (PFU)/ml, and its titer was measured after following incubation at different temperatures (30 °C, 40 °C, 50 °C, 60 °C, 70 °C, and 80 °C) for 1 h. The phage was then added to SM buffer (PH1845; Overview Phygene) at different pH ( pH range 2–11), and the titer was determined again after incubation for 1 h. Three biological replicates were performed for each experiment. These experiments allowed us to analyze the stability of the phage at different temperatures and pH values.

To evaluate the host range of *Aeromonas* phage vB AhM-LH, we conducted Spot assays tests [28]. In this procedure, 100 μl of bacterial overnight culture was mixed with semi-solid agar medium and the agar mixture spread evenly over a solid plate. Next, 5 μl of *Aeromonas* phage vB AhM-LH solution at varying dilutions was dropped onto the plates. The plates were then dried and transferred to an incubator for further incubation [28].

### In vitro antibacterial test

An in vitro assay was performed to assess the antibacterial efficacy of *Aeromonas* phage vB AhM-LH. *Aeromonas* phage vB AhM-LH and *Aeromonas hydrophila* AH3.5 were mixed at MOIs of 10, 1 and 0.1, respectively, incubated for 10 min, and then shaken at 30 °C for 5 h. The OD600 value was then measured using a UV spectrophotometer every 30 min. The control group was treated with an equal volume of SM buffer and shaken for 5 h. The experiment was performed in three biological replicates [29, 30].

### Phage genome extraction, sequencing and comparative genomics

The purified phage was extracted from DNA using a TIANamp Virus DNA/RNA Kit (DP315; Tiangen) [29]. Whole-genome shotgun (WGS) and next-generation sequencing (NGS) strategies were employed to construct libraries of different insert fragments. The Illumina NovaSeq sequencing platform (Illumina Inc., San Diego, CA, USA) was used for pair-end sequencing. The A5-MiSeq pipeline and SPAdes (v3.12.0) genome assembly algorithm were utilized to assemble the sequencing data without adapter sequences [31, 32]. Contigs were constructed by extracting sequences based on the sequencing depth of the spliced sequences and comparing them with the NT library on the National Center for Biotechnology Information (NCBI) databases (2018-09-05) using BLASTN [33]. Collinearity analysis of the contigs was performed using MUMmer software (v2.4.0) to determine their positional relationship and fill in the gaps [34]. Pilon software (v1.18) was applied to correct the results and obtain the final viral genome sequence [35]. Protein-coding genes were aligned using diamond software (v2.0.11), and the NCBI NR database (2018-11-22) was used for sequence alignment [36]. Diamond BLASTP was utilized to compare the protein sequence encoded by the gene with the protein sequence present in the database. The critical value for sequence alignment was set at 1e-6, and an optimal number of hits was chosen. To determine the function, the genome sequence, gene prediction, and non-coding RNA prediction information were integrated into a standard GBK (GenBank) format file. A circle diagram of the genome was then generated using cgview, followed by editing the diagram using Photoshop CS [37]. The complete genome of *Aeromonas* phage vB AhM-LH was sequenced and assembled by Personal Biotechnology Company (Shanghai, China) (SRS19190564).

The genome sequences of the 17 samples, after adjusting the starting point, were aligned using mafft software (v7.505) (https://mafft.cbrc.jp/alignment/software/). Subsequently, FastTree software (v2.1.11SSE3) (http://www.microbesonline.org/fasttree/) was employed to construct a phylogenetic tree using the maximum likelihood (ML) method, employing the generalized time-reversible (GTR) model and 1000 bootstrap replicates.

### Exposure of adult mosquitoes to phages

The *Aeromonas* phage vB AhM-LH concentrate was mixed with 5% sterile sugar water to achieve the desired concentration. For the sterile sugar water group (control), an equal volume of PBS buffer was added. Mosquitoes fed orally with bacteriophage were raised in sterilized paper cups, with 15–20 mosquitoes per cup. The mosquitoes fed on sterile cotton balls soaked with a mixture of 5% glucose water and phage. The cotton balls were replaced every 24 h for 4 days.

### Exposure of larvae to *Aeromonas hydrophila*

Mosquito larvae were reared in autoclaved water in containers, with four egg rafts per 2 l of water. Once the eggs hatched, *Aeromonas hydrophila* AH 3.5, isolated from the intestinal tract of DR strain female mosquitoes, was inoculated into LB medium. The culture was then expanded to OD600 = 1–1.5. The bacteria were then centrifuged at 12,000 *g* and resuspended in sterile PBS; this centrifugation/resuspension step was repeated twice more. The 100-ul bacterial suspension was mixed with autoclaved 0.15 g mouse feed powder every 24 h and fed to the mosquito larvae of the test group until pupation. The conventional feeding group was fed only germ-free mouse feed. After emergence, the experimental group was orally fed with phage, as described in section Phage genome extraction, sequencing and comparative genomics, while the mosquitoes in the control group were fed with sterile sugar water.

### Quantitative real-time PCR assays to detect *Aeromonas hydrophila*

To evaluate the abundance of *Aeromonas hydrophila* in the midguts of female mosquitoes within the studied population, the midguts of female mosquitoes that had not ingested a blood meal were dissected after being orally exposed to *Aeromonas* phage vB AhM-LH for 4 days in a sterile environment. Prior to dissection the mosquito was rinsed three times with 75% ethanol to remove surface bacteria, followed by three rinses with sterile PBS to remove the residual ethanol. Autoclaved anatomical forceps were used to dissect the midgut and place it in sterile 1.5-ml EP tubes, with each tube containing eight intestines. DNA was extracted using a Tianamp Micro DNA kit (DP316; Tiangen). Primers AH V4 F (5ʹ-GGCGGTTGGATAAGTTAGATGT-3ʹ) and AH V4 R (5ʹ-GCACCTGAGCGTCAGTCTT-3ʹ) were designed based on the 16S ribosomal DNA (rDNA) V4 region sequence of the *Aeromonas hydrophila* genome [9]. The β-actin gene was used as an internal control for real-time fluorescence quantitative PCR (qPCR), with primers β-actin-F (5ʹ-TGCGTGACATCAAGGAGAAGC-3ʹ) and β-actin-R (5ʹ-CCATACCCAAGAAGGAAGGCT-3ʹ) [38]. The reaction system consisted of 5 μl of 2× EvaGreen qPCR MasterMix-No Dye (Applied Biological Materials, Richmond, BC, Canada), 4 μl of midgut DNA template (approx. 400 ng) and 0.5 μL (10 μM) of each upstream and downstream primer. The reaction conditions were 95 °C for 300 s, followed by 40 cycles of 95 °C for 15 s and 60 °C for 60 s.

### Bioassays of mosquito susceptibility to deltamethrin

To assess changes in deltamethrin susceptibility levels of mosquitoes after oral feeding with *Aeromonas* phage vB AhM-LH, we followed the standard operating procedure for testing insecticide susceptibility of adult mosquitoes in WHO bottle bioassays [39]. A clean and dry 250-ml wide-mouth reagent bottle was prepared and 1 ml of deltamethrin, which approximated the LC50 (LC50: DR mosquitoes = 100 mg/l, DS mosquitoes = 0.25 mg/l, mosquitoes from the field = 12.5 mg/l), was added. An equal volume of acetone solution (≥ 99.9%) (270725; Sigma-Aldrich) was added to the negative control group bottle. Each bottle containing acetone was shaken to evenly distribute the solvent, then wrapped in tin foil and placed in a fume hood away from light where the fan was turned on to dry for 2 h to ensure the dissipation of any residual smell. The reagent bottle was then positioned vertically. Then, 20–25 female mosquitoes aged 3–5 days post-eclosion were placed in the bottle, and the mortality rate was determined every 15 min for 2 h. A mosquito was considered dead if it was able to lie on its back and move its legs and wings but unable to take off. It is important to note that in this bioassay the mortality rate in the acetone group should not exceed 20% [39].

### Statistical analysis

The data were expressed as the mean ± standard error of the mean (SEM). We used t-tests to assess: (i) differences in deltamethrin sensitivity levels among DR female mosquitoes; (ii) variations in intestinal *Aeromonas hydrophila* abundance between female mosquitoes from the field and DR female mosquitoes; and (iii) variations in *Aeromonas hydrophila* abundance and deltamethrin sensitivity levels within the intestinal tract of female mosquitoes from the field after *Aeromonas* phage vB AhM-LH treatment. Analysis of variance (ANOVA) was utilized to compare: (i) the difference in titers between different MOI of *Aeromonas* phage vB AhM-LH; (ii) variations in *Aeromonas hydrophila* abundance in the intestinal tract of DR female mosquitoes after treatment with varying concentrations of *Aeromonas* phage vB AhM-LH; and (iii) differences in *Aeromonas hydrophila* abundance and deltamethrin sensitivity levels in DS female mosquitoes after *Aeromonas* phage vB AhM-LH treatment. Gene expression was quantified using the 2^−ΔΔCt^ method [40] and statistically analyzed in GraphPad Prism 9.4 software (GraphPad Inc., La Jolla, CA, USA). A significance level of *P* < 0.05 was considered statistically significant.

## Results

### Isolation of *Aeromonas hydrophila* phage and transmission electron microscopy

**Fig. 1:**
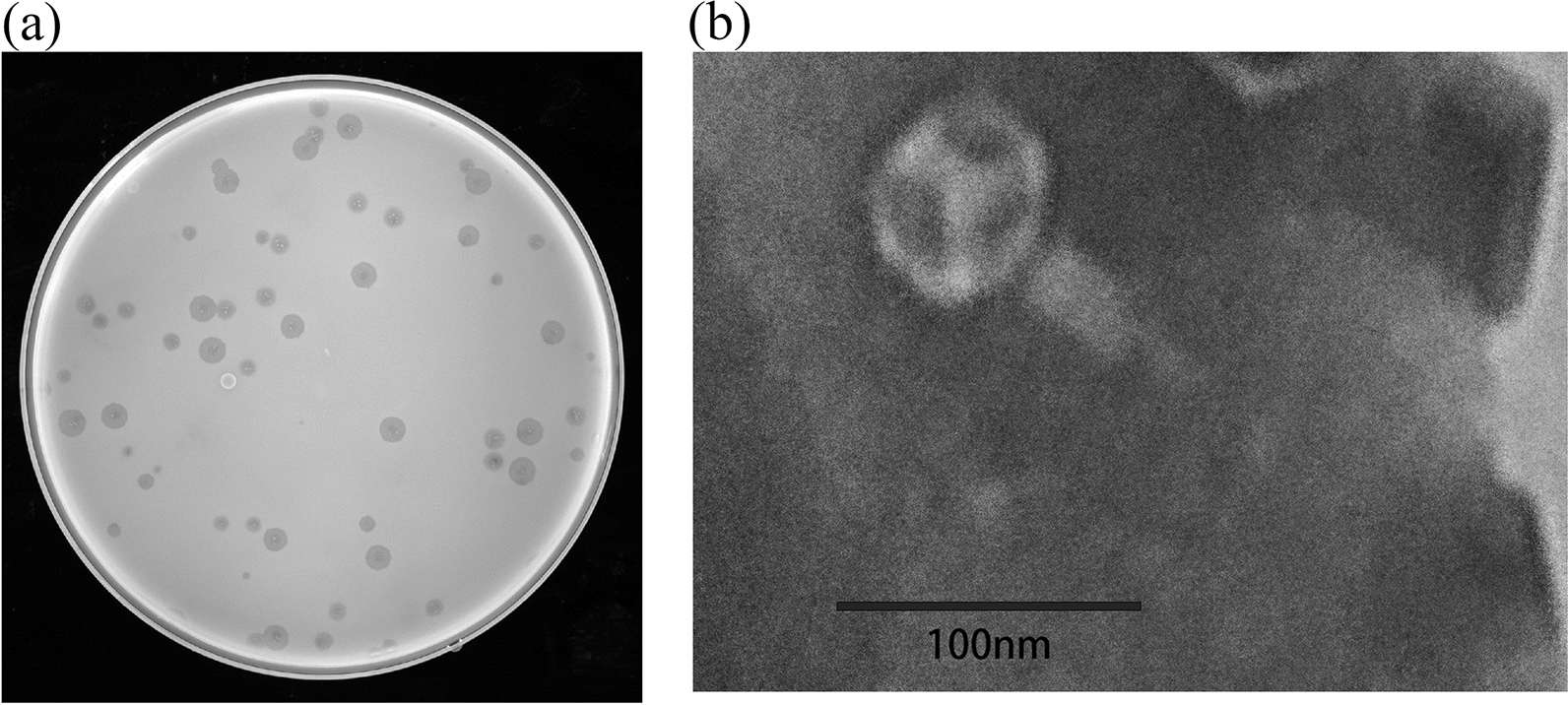
**a** Plaques formed by *Aeromonas* phage vB AhM-LH. **b** Morphology of purified *Aeromonas* phage vB AhM-LH under the transmission electron microscope

An *Aeromonas hydrophila* phage was isolated from a river in Nanjing, China, using the double-layer agar plate method. This phage was subsequently found to be able to form transparent vacuoles in *Aeromonas hydrophila* on LB plates (Fig. 1a). The electron microscopy study revealed that the head was ortho icosahedral in shape and that the phage had a tail (Fig. 1b). The phage was identified as belonging to the *Myoviridae* family in the order *Caudovirales*, and was named *Aeromonas* phage vB AhM-LH.

### Phage biological characteristics and in vitro antibacterial experiments

**Fig. 2:**
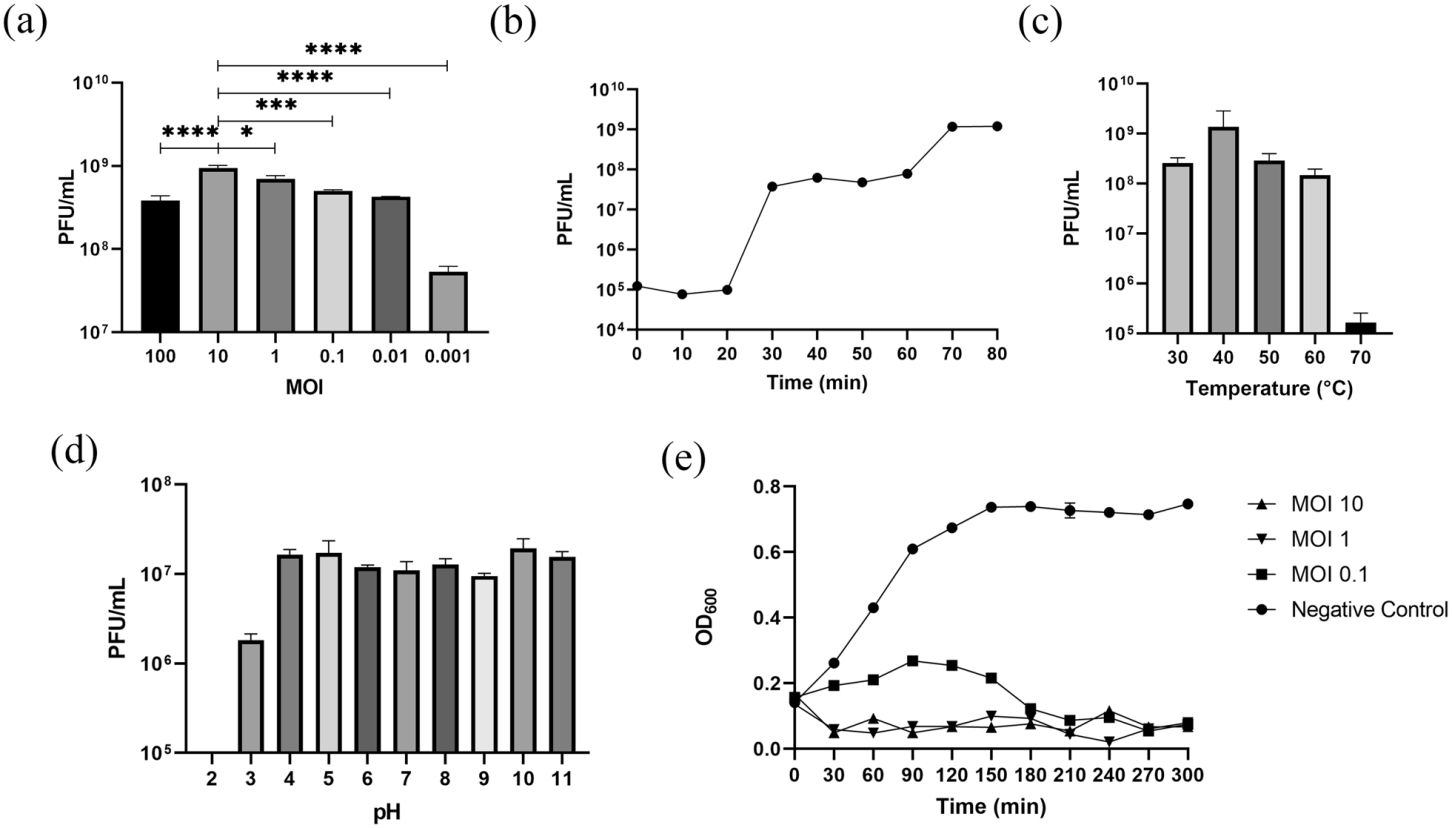
Biological characteristics of *Aeromonas* phage vB AhM-LH. **a** Phage titers at MOIs of 100, 10, 1, 0.1, 0.01, 0.001). **b** A one-step growth curve of phage LH at an MOI of 10. **c **Phage LH titer after incubation for 1 h at 30 °C, 40 °C, 50 °C, 60 °C, 70 °C and 80 °C. **d** Stability of phage LH at different pH (range pH 2 to 11). **e** In vitro antibacterial experiment of phage LH at MOIs of 10, 1 and 0.1 showing inhibition of growth of host bacteria by phage LH (shown as change in OD600). Data are presented as the mean ± SE of 3 biological replicates and 3 technical replicates. Asterisks in** a** indicate significant differences at *P ≤ 0.05, **P < 0.01, ***P < 0.001 and ****P < 0.0001. MOI, Multiplicity of infection; OD600, optical density at 600 nm wavelength; PFU, plaque forming units; phage LH, *Aeromonas* phage vB AhM-LH; SE, standard error

The optimal MOI for *Aeromonas* phage vB AhM-LH was determined to be 10 (Fig. 2a). The one-step growth curve results showed that at an MOI of 10, the incubation period of *Aeromonas* phage vB AhM-LH was 20 min, the burst period was 50 min and the burst volume was 118 (Fig. 2b). *Aeromonas* phage vB AhM-LH remained stable at temperatures between 30 °C and 60 °C; however, its titer significantly decreased at 70 °C and the phage became completely inactive at 80 °C (Fig. 2c). Additionally, *Aeromonas* phage vB AhM-LH exhibited higher activity across a pH range of 4 to 11 (Fig. 2d).

The results from the in vitro antibacterial experiments demonstrated that *Aeromonas* phage vB AhM-LH effectively inhibited the growth of host bacteria at MOIs of 10, 1 and 0.1 (Fig. 2e). Host spectrum analysis revealed that *Aeromonas* phage vB AhM-LH lysed seven strains of *Aeromonas hydrophila* isolated from the intestines of resistant female mosquitoes, but did not show lytic activity against *Escherichia coli* BL21 and three other bacteria isolated from the mosquito intestines (Table 1).

**Table 1:** Host range of *Aeromonas* phage vB AhM-LH

Bacteria	Lysis by LH	Source
*Aeromonas hydrophila* AH1	Yes	Laboratory isolation
*Aeromonas hydrophila* AH2	Yes	Laboratory isolation
*Aeromonas hydrophila* AH3.2	Yes	Laboratory isolation
*Aeromonas hydrophila* AH3.3	Yes	Laboratory isolation
*Aeromonas hydrophila* AH3.5	Yes	Laboratory isolation
*Aeromonas hydrophila* AH6	Yes	Laboratory isolation
*Aeromonas hydrophila* AH7	Yes	Laboratory isolation
*Asaia*	No	Laboratory isolation
*Serratia oryzae*	No	Laboratory isolation
*Enterococcus *sp.	No	Laboratory isolation
*Escherichia coli* BL21	No	Shanghai Bioresource Collection Center

*LH Aeromonas* phage vB AhM-LH

### Whole-genome sequencing and comparative genomics

**Fig. 3:**
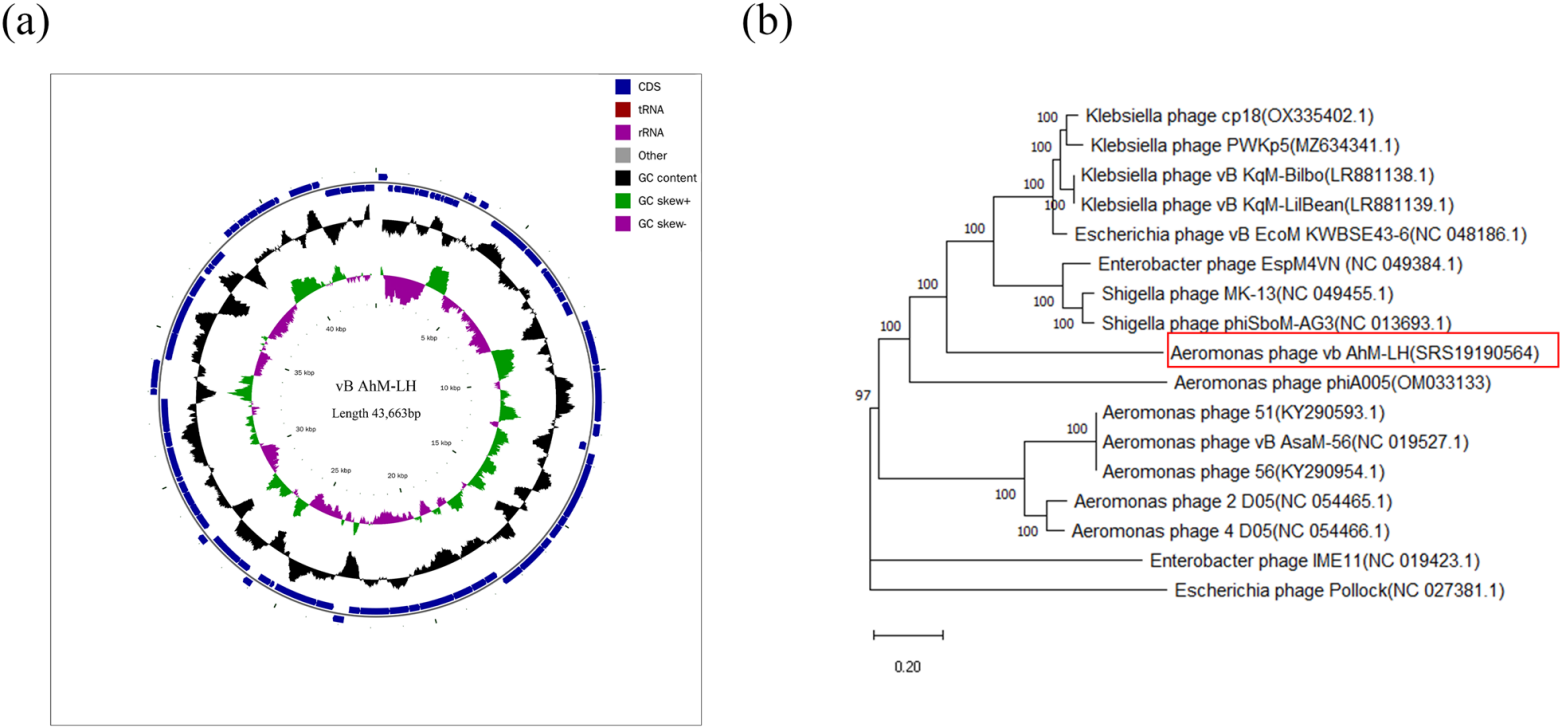
**a** *Aeromonas* phage vB AhM-LH genome circle diagram. **b** Neighbor-joining phylogenetic tree illustrating the relationship between *Aeromonas* phage vB AhM-LH and phages as identified in the NCBI nucleotide BLAST search (BLASTN; 2022-12-26). Each branch represents a species, and the length of the branch indicates the evolutionary distance between the species, reflecting the degree of difference between them. The number (bootstrap value) marked on the node indicates the credibility of clustering of all species in the clade into a single clade

Analysis of the genome of *Aeromonas* phage vB AhM-LH revealed that it is a linear double-stranded DNA with a total length of 43,663 bp. There are 81 open reading frames, which make up 92.3% of the whole genome (Table 2). A comparison at the non-redundant (NR) database showed that the *Aeromonas* phage vB AhM-LH genome has the capacity to encode 21 hypothetical proteins, 24 proteins with known functions and two proteins with unknown functions. The known functional proteins comprise 11 phage structural proteins, 12 replication-transcription-related proteins and one bacterial lysis-related protein (Table 3). The *Aeromonas* phage vB AhM-LH genome circle diagram is shown in Fig. 3a. Importantly, there are no genes encoding integrase-related proteins in the *Aeromonas* phage vB AhM-LH genome, suggesting that *Aeromonas* phage vB AhM-LH has the potential for application in phage therapy.

**Table 2:** Statistics for open reading frame prediction

ORF properties	Statistics
ORF number	81
ORF total length	40,299 bp
ORF density	1.855 genes per kb
Longest ORF length	1797 bp
ORF average length	497.52 bp
Intergenic region length	3364 bp
ORF/Genome (coding percentage)	92.30%
Intergenic length/Genome	7.70%
GC content in ORF region	52.00%
GC content in the intergenic region	46.97%

ORF Open reading frame

**Table 3:** Non-redundant annotation of protein-coding genes

ORF	Predicted functions	Start (bp)	End (bp)	Length (bp)	Direction
ORF1	Hypothetical protein	584	817	77	Forward
ORF2	Hypothetical protein	817	1269	150	Forward
ORF3	Hypothetical protein	1395	1730	111	Forward
ORF4	DNA-binding domain protein	2260	2577	105	Forward
ORF5	Hypothetical protein	2994	3245	83	Forward
ORF6	Terminase large subunit	4013	5434	473	Forward
ORF7	DNA-binding protein	5456	6133	225	Forward
ORF8	Hypothetical protein	6298	6426	42	Forward
ORF9	Terminase small subunit	6419	7024	201	Forward
ORF10	putative phosphoadenosine phosphosulfate reductase	8730	9830	366	Forward
ORF11	P-loop containing nucleoside triphosphate hydrolase	10,062	11,615	517	Forward
ORF12	Protein of unknown function	12,632	12,985	117	Forward
ORF13	P-loop containing nucleoside triphosphate hydrolase	12,978	14,774	598	Forward
ORF14	Nuclease	15,088	15,549	153	Forward
ORF15	Hypothetical protein	15,629	15,970	113	Forward
ORF16	RecT protein	15,967	16,905	312	Forward
ORF17	Putative exonuclease	16,905	17,597	230	Forward
ORF18	Tail fiber assembly protein	17,623	18,045	140	Forward
ORF19	Protein of unknown function	18,545	19,213	222	Forward
ORF20	Baseplate protein J-like protein	19,210	20,376	388	Forward
ORF21	Hypothetical protein	20,376	20,735	119	Forward
ORF22	Hypothetical protein	20,735	21,382	215	Forward
ORF23	Hypothetical protein	21,375	22,349	324	Forward
ORF24	Hypothetical protein	22,844	23,200	118	Forward
ORF25	Hypothetical protein	23,226	23,813	195	Forward
ORF26	Tail tape measure	23,813	25,258	481	Forward
ORF27	Hypothetical protein	26,288	26,704	138	Forward
ORF28	Tail sheath protein	26,705	27,817	370	Forward
ORF29	Hypothetical protein	27890	28,192	100	Forward
ORF30	Minor capsid protein	28,740	29,132	130	Forward
ORF31	Neck protein	29,113	29,586	157	Forward
ORF32	Head completion adaptor	29,579	29,971	130	Forward
ORF33	Major capsid protein	30,147	31,160	337	Forward
ORF34	Coil containing protein	31,661	32,773	370	Forward
ORF35	Hypothetical protein	32,900	33,106	68	Forward
ORF36	Hypothetical protein	33,124	34,014	296	Forward
ORF37	Minor capsid component	34,042	34,953	303	Forward
ORF38	Portal protein	34,940	36,253	437	Forward
ORF39	DNA methylase	36,290	37,018	242	Forward
ORF40	Hypothetical protein	37,171	37,377	68	Forward
ORF41	Hypothetical protein	37,532	38,044	170	Forward
ORF42	Hypothetical protein	38,742	39,041	99	Forward
ORF43	NTP-PPase-like protein	39,446	39,745	99	Forward
ORF44	Single strand binding protein	39,881	40,387	168	Forward
ORF45	Hypothetical protein	40,897	41,640	247	Forward
ORF46	Hypothetical protein	41,651	41,884	77	Forward
ORF47	Hypothetical protein	42,475	42,843	122	Forward

ORF Open reading frame

The phylogenetic analysis revealed that *Aeromonas* phage vB AhM-LH is a newly discovered phage (Fig. 3b). It shares a close genetic relationship with *Aeromonas* phage phiA005, as well as with other *Aeromonas* phages, such as 51, 56 vB AsaM-56, 2 D05 and 4 D05. Additionally, it is distantly related to *Enterobacter* phages EspM4VN and IME11, *Shigella* phages phiSboM-AG3 and MK-13, *Escherichia* phage vB EcoM Kwbse43-6 and *Klebsiella* phages vB KqM-LilBean, KqM-Bilbo, PWKp5 and cp18.

### Effect of oral feeding of phages on deltamethrin resistance in mosquitoes

**Fig. 4:**
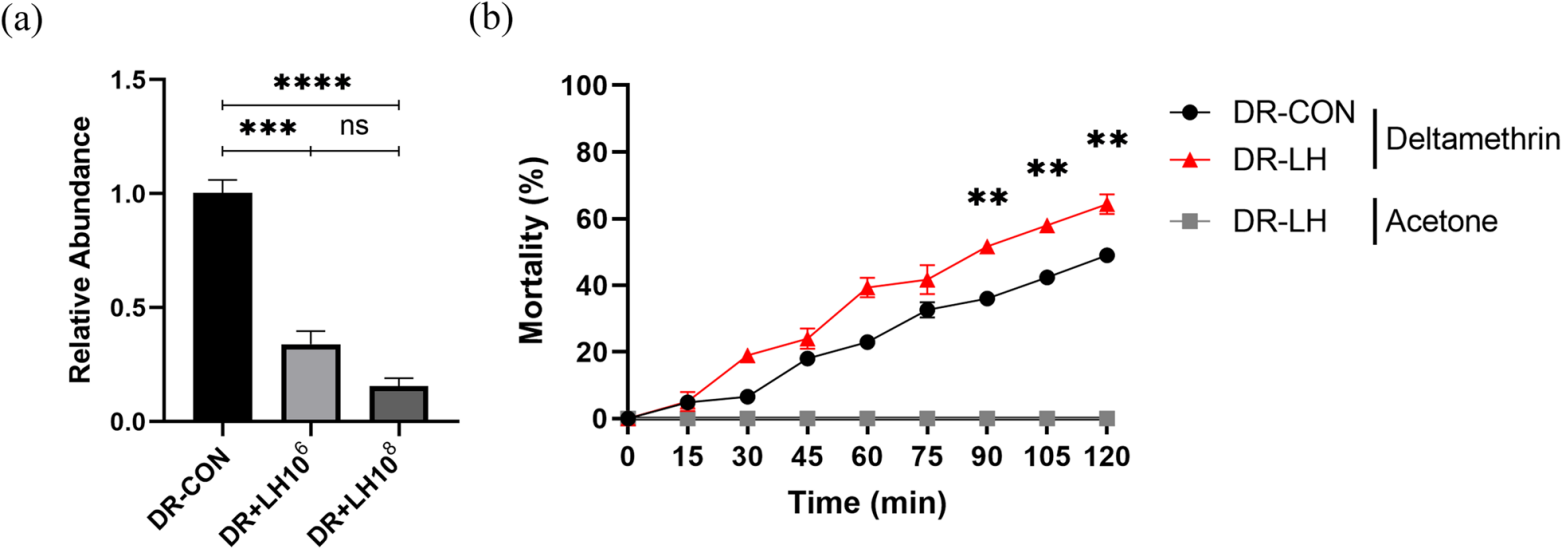
**a** Relative abundance of *Aeromonas hydrophila* in the gut of DR female mosquitoes. **b** Mortality of *Aeromonas hydrophila* after exposure to deltamethrin for varying lengths of time in DR female mosquitoes. Data are presented as the mean ± SE of 3 biological replicates and 3 technical replicates. Asterisks indicate significant differences at **P < 0.01, ***P < 0.001 and ****P < 0.0001. CON, Control; DR, Deltamethrin resistant; LH10^6^ and LH10^8^, Concentration of *Aeromonas* phage vB AhM-LH in sugar water (PFU/ml); ns, not significant; PFU, plaque-forming units

To investigate the impact of *Aeromonas* phage vB AhM-LH on *Aeromonas hydrophila* abundance in the DR mosquito midgut, newly emerged DR female mosquitoes were orally fed a sucrose diet containing phage at 10^6^ and 10^8^ PFU/ml, while the control group received sterile sugar water. After 4 days, qPCR analysis was used to measure the relative abundance of *Aeromonas hydrophila* in the midgut of female mosquitoes. The results showed that in comparison with the control group, the abundance of *Aeromonas hydrophila* in the midgut of females in the phage-fed groups was reduced by 66% (*F*_(2/6)_ = 76.85, *P* = 0.0002) and 85% (*F*_(2/6)_ = 76.85, *P* < 0.0001) in the DR+10^6^ and DR+10^8^ groups, respectively (Fig. 4a). The abundance of *Aeromonas hydrophil*a in the DR+10^8^ group was 19% lower than that in the DR+10^6^ group (*F*_(2/6)_ = 76.85, *P* = 0.0935). These results demonstrated that oral administration of *Aeromonas* phage vB AhM-LH significantly decreased the abundance of *Aeromonas hydrophila* in the midgut of DR female mosquitoes, with the effect becoming more pronounced at higher phage concentrations. The concentration of 10^8^ PFU/ml was selected for subsequent experiments because it effectively reduced the abundance of intestinal *Aeromonas hydrophila*. We also compared the resistance levels of adult mosquitoes orally fed with 10^8^ PFU/ml phage to those of adult mosquitoes fed sterile sugar water using a WHO bottle test. The results of this bioassay showed that following exposure to deltamethrin, mortality in the phage group was significantly higher than that in the control group at 90, 105 and 120 min of exposure, with 15% (*t*_(4)_ = 5.875, *P* = 0.0042), 16% (*t*_(4)_ = 7.431, *P* = 0.0018) and 15% (*t*_(4)_ = 4.904, *P* = 0.0080) increased mortality, respectively (Fig. 4b).

### DS mosquito *Aeromonas hydrophila* addition and elimination experiment

**Fig. 5:**
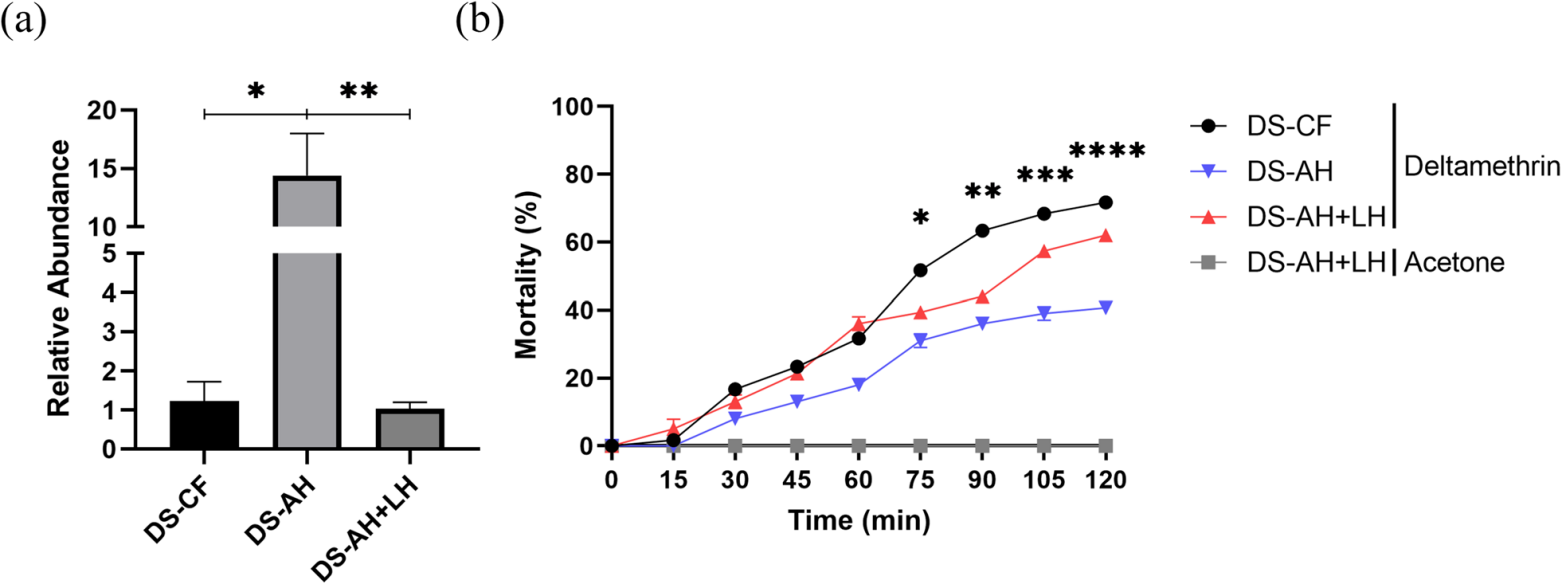
**a** Relative abundance of *Aeromonas hydrophila* in the gut of DS female mosquitoes**. b** Mortality of *Aeromonas hydrophila* after exposure to deltamethrin for varying lengths of time in DS female mosquitoes. Data are presented as the mean ± SE of 3 biological replicates and 3 technical replicates. Asterisks indicate significant differences at *P ≤ 0.05, **P < 0.01, ***P < 0.001 and ****P < 0.0001. DS, Deltamethrin sensitive; DS-AH, treatment group consisting of DS larvae colonized with *Aeromonas hydrophila*; DS-AH+LH, treatment group consisting of DS larvae colonized with *Aeromonas hydrophila* and orally fed *Aeromonas* phage vB AhM-LH; DS-CF, deltamethrin-sensitive treatment group (conventional feeding); SE, standard error

To investigate the impact of *Aeromonas* phage vB AhM-LH on deltamethrin resistance in mosquitoes, larvae from the DS strain were provided with standard food supplemented with *Aeromonas hydrophila* (sample DS-AH), while the control larvae (DS) received standard food without the addition of bacteria (DS-CF). After emergence, the DS-AH adults had access an oral source of sugar containing the phage (DS-AH-LH) while other group fed on sterile sugar water. The qPCR analysis revealed that the abundance of *Aeromonas hydrophila* in the DS-AH group was 13.1-fold higher than that in the DS-CF group after 4 days (*F*_*(2/6)*_ = 13.38, *P* = 0.0103) (Fig. 5a). In the DS-AH+LH group, the abundance of *Aeromonas hydrophila* in the intestine decreased by 92.8% compared with that in DS-AH group (*F*_*(2/6)*_ = 13.38, *P* = 0.0096), approaching levels seen in the DS-CF group (Fig. 5a). These findings suggested that administering *Aeromonas* phage vB AhM-LH orally to female mosquitoes post-emergence could significantly decrease the number of *Aeromonas hydrophila* colonized in the intestines of larvae. Results of the WHO bottle bioassay showed that the mortality of the DS-AH group was lower than that of the DS-CF group after exposure to deltamethrin for 75, 90, 105 and 120 min, with reductions in mortality of 20.6% (*F*_(2/6)_ = 42.93, *P* = 0.0002), 27.3% (*F*_(2/6)_ = 124.0, *P* < 0.0001), 29.3% (*F*_(2/6)_ = 71.43,* P* < 0.0001), and 31.0% (*F*_(2/6)_ = 135.9, *P* < 0.0001), respectively. The DS-AH+LH group exhibited a mortality that was 18.3% (*F*_(2/6)_ = 71.43, *P* = 0.0008) higher than that in the DS-AH group at 105 min and 22.0% (*F*_(2/6)_ = 135.9, *P* < 0.0001) higher at 120 min of exposure (Fig. 5b).

These findings demonstrated that the concentration of *Aeromonas hydrophila* in the larval growth environment significantly influences its abundance in the intestinal tract of adults. Additionally, the resistance of female mosquitoes to deltamethrin increases with the abundance of *Aeromonas hydrophila* in the intestine, but can be decreased through oral administration of *Aeromonas* phage vB AhM-LH.

### Application of* Aeromonas* phage vB AhM-LH to *Culex pipiens pallens* from the field

To evaluate the potential of *Aeromonas* phage vB AhM-LH to control vector resistance, we collected mosquito larvae from a ditch near a residential area in Nanjing, China. The DNA barcode identification results confirmed that the species was *Cx. pipiens pallens* (Additional file 1: Sequence S1).

**Fig. 6:**
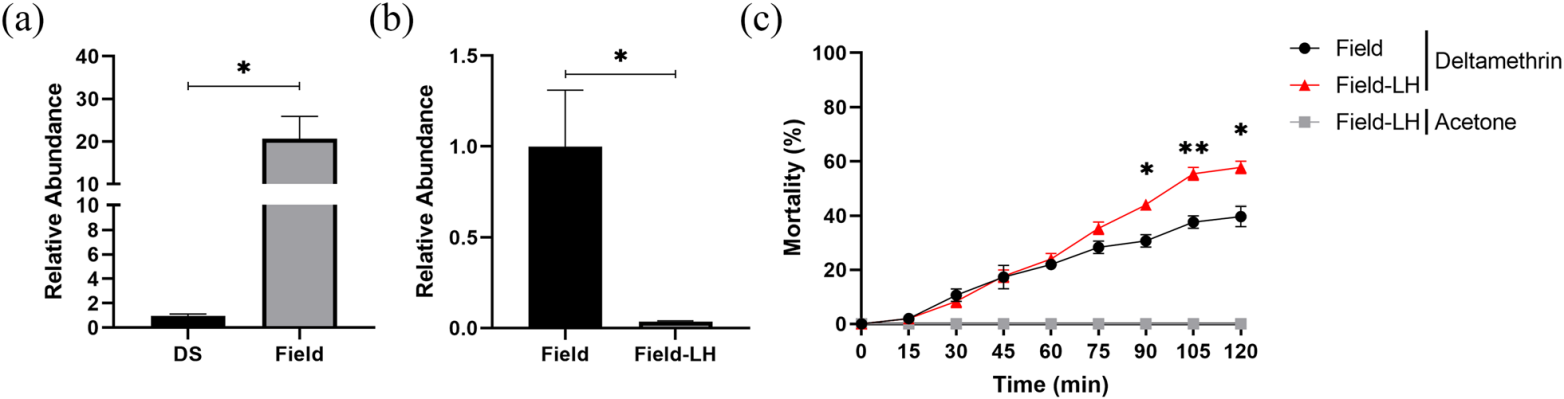
**a** Comparison of the abundance of *Aeromonas hydrophila* in the intestines of Field and DS female mosquitoes. Statistical values were calculated relative to the DS group. **b** Relative abundance of *Aeromonas hydrophila* in the intestinal tract of female mosquitoes collected in the field. **c** Mortality after exposure to deltamethrin in female mosquitoes collected in the field. Data are presented as the mean ± SE of 3 biological replicates and 3 technical replicates. Asterisks indicate significant differences at *P ≤ 0.05. DS, Deltamethrin sensitive; Field, group consisting of female mosquitoes collected in the field; Field-LH, treatment group consisting of female mosquitoes collected in the field orally fed with *Aeromonas* phage vB AhM-LH at 10^8^ PFU/ml; SE, standard error

The abundance of *Aeromonas hydrophila* in the intestines of DS mosquitoes raised in the laboratory and in female *Cx. pipiens pallens* collected in the field was compared using qPCR. This comparison was performed 4 days after emergence. The results revealed that the abundance of *Aeromonas hydrophila* in the intestinal tract of female mosquitoes collected in the field was 19.7-fold (*t*_(4)_ = 3.758, *P* = 0.0198) higher than that of laboratory-reared DS females (Fig. 6a). Subsequently, newly emerged female mosquitoes collected in the field were orally fed with *Aeromonas* phage vB AhM-LH at 10^8^ PFU/ml (Field-LH group). The control group was orally fed with sterile sugar water (Field group). The results indicated a significant decrease in the abundance of *Aeromonas hydrophila* in the midgut of females from the Field group fed with phages; specifically, there was a 96.3% (*t*_(4)_ = 3.097, *P* = 0.0363) decrease in *Aeromonas hydrophila* abundance in the Field-LH group compared with the Field group (Fig. 6b). The results from the WHO bottle bioassay showed that the Field-LH group had increased mortality rates at 90, 105 and 120 min of exposure, compared with those of *Cx. pipiens pallens* collected in the field that were orally fed with sterile sugar water. The rates increased by 13.3% (*t*_(4)_ = 4.339, *P* = 0.0123), 17.7% (*t*_(4)_ = 5.354, *P* = 0.0059), and 18.0% (*t*_(4)_ = 4.070, *P* = 0.0152) at 95, 105 and 120 min of exposure, respectively (Fig. 6c).

## Discussion

In this study, a novel phage, *Aeromonas* phage vB-AhM-LH, was isolated and morphologically characterized for the first time from *Aeromonas hydrophila*, a mosquito intestinal symbiont associated with insecticide resistance [9]. Biological characterization and genomic analysis revealed that *Aeromonas* phage vB-AhM-LH shows promise as a bioantimicrobial agent. *Aeromonas* phage vB AhM-LH exhibited favorable stability in terms of temperature and pH, maintaining viability in the mosquito intestinal pH range of 8.5–9.5 [41], and demonstrated effective in vitro bacteriostatic activity and host specificity. The results from experiments conducted on mosquitoes suggested that *Aeromonas* phage vB AhM-LH could be a potential tool to manage insecticide resistance transmitted by mosquitoes.

The intestinal microorganisms of adults primarily originate from the environment where the larvae inhabit [42]. When pupae emerge, they lose most of their intestinal microorganisms, and some bacteria from the larval stage are transferred to adult mosquitoes [42, 43]. The microbiome of adult mosquitoes is simpler compared with that of larvae and is more susceptible to manipulation by phages. Our findings demonstrated that the newly isolated phage identified in our study could effectively suppress the relative abundance of *Aeromonas hydrophila* in the gut of female *Cx. pipiens pallens* and reduce its resistance to deltamethrin.

Recent research has shown that the proliferation of phages in the body of house flies can alter the intestinal flora and negatively impact the development of larvae [16]. Additionally, symbiotic microorganisms in the insect gut play a crucial role in regulating insect metabolism, enhancing food digestion, increasing waste excretion, protecting the host from enemies, developing resistance to toxins and degrading toxins into intermediates [44]. Therefore, the change in resistance phenotype observed in female mosquitoes might be a direct result of the reduced abundance of *Aeromonas hydrophila* or could be attributed to the imbalance in the intestinal flora of the adults. One limitation of our study is that we did not utilize 16S rRNA gene sequencing to explore the impact of *Aeromonas* phage vB AhM-LH on the intestinal microbial community of female mosquitoes.

DS female mosquitoes raised in the laboratory exhibit significantly lower resistance to deltamethrin compared with DR female mosquitoes [9]. Additionally, the content of *Aeromonas hydrophila* in the intestines of DS mosquitoes is considerably lower than that in DR mosquitoes [9]. The relationship between the abundance of *Aeromonas hydrophila* in the mosquito gut and the sensitivity levels of mosquitoes to deltamethrin can be demonstrated through this study. We conducted colonization and elimination experiments on DS mosquitoes and confirmed that changes in the abundance of *Aeromonas hydrophila* in the mosquito intestines directly affected mosquito resistance to deltamethrin. The axenic and gnotobiotic mosquito model is an emerging method to study the interaction between commensal bacteria and the host, because it eliminates the interference of the original intestinal flora [10]. However, despite our efforts to sterilize *Cx. pipiens pallens* egg valves using bleach and ethanol, we were only able to obtain sterile *Cx. pipiens pallens* larvae [45]. Unfortunately, during the colonization process, *Aeromonas hydrophila* proliferates in sterile water using larval feed, leading to the death of most of the sterile larvae. Moreover, it is difficult for larvae lacking bacteria to develop to the second instar, which limits the possibility of conducting further experiments.

While the results of our research suggested that changes in the abundance of *Aeromonas hydrophila* in the gut impact adult resistance to deltamethrin, the relationship between the abundance of *Aeromonas hydrophila* and the level of deltamethrin resistance still needs to be established. Our investigation uncovered a significant disparity in deltamethrin resistance between field-collected and DS female mosquitoes, which was found to be greater than the difference in the abundance of *Aeromonas hydrophila* in the adult midgut. A previous study demonstrated that target mutations and increased enzymatic activity are the primary mechanisms of insecticide resistance in *Cx. pipiens* [46]. The mechanisms by which *Cx. pipiens pallens* develops resistance to deltamethrin varies depending on the intensity of insecticide selection pressure [47], with target site mutations contributing significantly under low pesticide selection pressure and metabolic resistance playing a greater role under high pesticide selection pressure [47]. In the field, mosquitoes are exposed to insecticides at varying concentrations and durations, resulting in the synergistic action of multiple resistance mechanisms [48]. Previous research has identified widespread target gene mutations in *Cx. pipiens* in mosquito control operation areas [48, 49]. Recent studies have indicated that symbiotic bacteria enhance mosquito resistance by directly breaking down the insecticide and increasing their metabolic enzyme activity [6, 9]. Nonetheless, the impact of symbiotic bacteria on target site susceptibility has not been investigated. Therefore, further research is needed to explore the role of bacteria in the midgut for target site sensitivity, and to investigate how adult resistance to deltamethrin insecticide influences the colonization of *Aeromonas hydrophila* in the midgut of mosquitoes.

*Cx. pipiens pallens* populations are frequently found in stagnant water sources, such as sewage pools, cesspools and sewers [49]. This habitat preference correlates with the prevalence of *Aeromonas hydrophila* [50], potentially explaining the elevated levels of *Aeromonas hydrophila* found in the intestines of the mosquitoes collected in the field in the present study. Additionally, *Aeromonas hydrophila* has been detected in *Culex* mosquito specimens collected from different regions [50]. Interestingly, despite the mosquitoes collected in the field having a much higher abundance of *Aeromonas hydrophila* in their intestinal tracts compared with the laboratory-raised mosquitoes, both sensitive and resistant mosquitoes, *Aeromonas* phage vB AhM-LH still effectively reduced the intestinal abundance of *Aeromonas hydrophila* in *Cx. pipiens pallens* collected in the field, suggesting that there might be a similarity in the intestinal commensal bacteria among the same mosquito species [42]. A previous study detected *Aeromonas* in different *Aedes aegypti* populations in Brazil [51]. Additionally, *Thorsellia*, *Wolbachia*, *Massilia* and *Acinetobacter * spp. have been found in *Anopheles gambiae* collected from various locations in Burkina Faso [52]. This microbial similarity facilitates the utilization of phages to aid the management of vector resistance. Further verification should be conducted using *Cx. pipiens pallens* in field or other mosquito species from different regions.

## Conclusions

In this study, we utilized a bacteriophage to investigate deltamethrin resistance in adult mosquitoes. Our findings revealed that the alteration in host bacteria mediated by phages can impact the resistance phenotype of adult mosquitoes. These results highlight the effectiveness of phage therapy in manipulating the gut microbiota of mosquito vectors, offering a promising approach to manage vector resistance.

### Supplementary Information

**Additional file 1: Sequence S1.** COI Sequence of *Culex pipiens* *pallens *from the field*.*

## Data Availability

All data generated or analyzed in this study are included in this published article.
